# Lawrence's Improved Portable Blow-Pipe

**Published:** 1848-10

**Authors:** 


*Lawrence's Improved Portable Blow-Pipe.*—I have just received from Mr. Henry Lawrence, now temporarily resident in Allentown, Pa., a most beautiful little bellows blow-pipe, recently invented by him, which, for the perfection of its construction, the ease and facility with which it is used, its extreme portability, and though last, not least, its great cheapness compared with all others now in use, cannot, I think, fail highly to recommend it to dentists, and to any other persons whose occupation requires the use of such an instrument.

The whole machine is but about nine inches long, eight inches wide, and six inches deep; and even these small dimensions may be contracted if greater portability be desired, by removing certain portions of it; for every part of the blow-pipe, excepting the bellows, may be taken in pieces and re-united in one minute's time.

It is, in brief, constructed as follows: A small double bellows is made, with a treadle for the foot fixed horizontally over it, to one end of which a hinge is attached, the other being rendered stationary by a little hasp and staple. Instead of weights, the bellows is made to rise and fall upon the application of the foot to the treadle, by two springs of coiled brass wire, properly attached to the machine. The air passes through a long flexible tube, with a brass jet attached, by means of which the operator obtains a facility in the management of the flame, which, in my opinion, alone renders this little instrument superior to any I have yet seen.

I feel assured, Messrs. Editors, that few dentists who have hitherto contented themselves with the common mouth blow-pipe, will fail to supply their laboratories with this simple and most effectual little machine, after they shall have witnessed the ease and certainty with which it performs its appropriate work. Its price also—which I presume will not exceed one-third that of the ordinary bench blow-pipe—taken in connexion with the superiority of its operation, must, I think, speedily induce a large majority of our dentists to adopt it so soon as it shall be offered for sale.—*Dental News Letter.*

